# Green Synthesis of Antibacterial CuO Nanoparticles Based on the Synergy Between *Cornu aspersum* Snail Mucus and Ascorbic Acid

**DOI:** 10.3390/molecules30020291

**Published:** 2025-01-13

**Authors:** Maria Todorova, Angelina Kosateva, Ventsislava Petrova, Bogdan Ranguelov, Stela Atanasova-Vladimirova, Georgi Avdeev, Ivanka Stoycheva, Emiliya Pisareva, Anna Tomova, Lyudmila Velkova, Aleksandar Dolashki, Pavlina Dolashka

**Affiliations:** 1 Institute of Organic Chemistry with Center for Phytochemistry, Bulgarian Academy of Sciences, 1113 Sofia, Bulgaria; krasimirova_m@yahoo.com (M.T.); angelina.kosateva@orgchm.bas.bg (A.K.); stoycheva@orgchm.bas.bg (I.S.); lyudmila.velkova@orgchm.bas.bg (L.V.); or adolashki@yahoo.com (A.D.); 2 Center of Competence “Clean Technologies for Sustainable Environment—Water, Waste, Energy for Circular Economy”, 1000 Sofia, Bulgaria; 3 Faculty of Biology (SU-BF), Sofia University “St. Kliment Ohridski”, 1504 Sofia, Bulgaria; vpetrova@biofac.uni-sofia.bg (V.P.); episareva@uni-sofia.bg (E.P.); aatomova@biofac.uni-sofia.bg (A.T.); 4 Institute of Physical Chemistry “Rostislav Kaishev”, Bulgarian Academy of Sciences, 1113 Sofia, Bulgaria; rangelov@ipc.bas.bg (B.R.); statanasova@ipc.bas.bg (S.A.-V.); g_avdeev@ipc.bas.bg (G.A.)

**Keywords:** antimicrobial inhibitor, ascorbic acid, copper nanoparticles, snail *Cornu aspersum*

## Abstract

Many biologically active compounds have been identified in the mucus of the garden snail *Cornu aspersum*, which are effective in the treatment of several diseases such as cancer, ulcers, wounds, etc. The incorporation of these compounds into the green synthesis of copper nanoparticles (CuONPs-Muc) was demonstrated in our previous study. Based on the synergistic effect of two reducing agents—*C. aspersum* snail mucus and ascorbic acid (AsA)—on CuSO_4_.5H_2_O, which also act as stabilizers of the resulting compound, a new method for the “green” synthesis of CuONPs-Muc is presented. Using two reducing agents has several advantages, such as forming spherical nanoparticles with a diameter of about 150 nm and reducing the formation time of CuONPs-Muc to 3 h. Analyses by ultraviolet–visible spectroscopy (UV-Vis) and Fourier transform infrared spectroscopy (FT-IR) show the formation of CuONPs-Muc, composed of a mixture of copper and copper oxide. This was confirmed by scanning electron microscopy combined with energy-dispersive spectroscopy (SEM/EDS) and X-ray diffraction (XRD). Another important advantage of CuONPs obtained by the new method with two reducing agents is the stronger inhibitory effect on the bacterial growth of some Gram-positive and Gram-negative bacterial strains, compared to CuONPs-Muc prepared with only one reducing agent, i.e., a fraction of mucus with an MW > 20 kDa.

## 1. Introduction

Metal nanoparticles (MNPs) have attracted the attention of many researchers with their easy and environmentally friendly production and their wide application in medicine, optics, biotechnology and photocatalysis, electronic circuits, and other fields [1,2,3,4,5]. Various approaches are used to obtain the nanoparticles, such as microwave irradiation [6], laser ablation [7], electrochemical reduction [8], arc discharge [9], microemulsion [10], physical and chemical reduction, etc. The most widely used method for the preparation of MNPs is a reduction in aqueous solution, characterized by a number of advantages such as quality and a high yield of particles, limited equipment requirements, and the simplicity of operation and control. Another method of preparing CuONPs by reducing Cu^2+^ ions with ascorbic acid in aqueous solution at different pHs of the solution has been presented, which affects the average size of CuONPs [11].

It is known that the chemical synthesis of nanoparticles often uses toxic and expensive chemicals that have an adverse effect on human health and the environment. Green synthesis, which is non-toxic and environmentally friendly, has been developed as the most appropriate approach to solving these problems [12,13]. The green synthesis of copper, silver, zinc, and gold nanoparticles (Cu-, Ag-, Zn-, and AuNPs) with plant extracts as reducing agents has been well studied [1,2,3]. The extract of green leaves from various plants, such as from the bark of *Cedrus deodara*, etc., is commonly used as a complexing agent for the safe and easy synthesis of CuONPs [2].

As a bioreducing agent of Cu, Ag, Au, Co, and Zn ions, the mucus of different species of snails has also been used, and a number of methods for obtaining AgNPs-SM from two land snails, *Lissachatina fulica* (Achatina) and *Hemiplecta distincta,* have been presented [14], as well as those for CuONPs from the garden snail *C. aspersum* [3].

Recently, environmentally pure synthesized biogenic substances from *Achatina fulica* snail mucus were studied by Mane et al., which showed strong anticancer activity against Hela cells and antibacterial and antifungal inhibition [15]. Moreover, copper oxide (SM-CuONC) and cobalt oxide (SM-Co_3_O_4_NC) bionanocomposites prepared by green synthesis produce antioxidant and anticancer activity in humans against breast cancer (MDA-MB-231), colon cancer (HCT-15), and cancer of the cervix (HeLa), etc. **[3,15,16]**.

One of the best-known land snails is the brown garden snail *Helix aspersa* (*C. aspersum*), which is a proven source of biologically active components, such as hyaluronic and glycolic acids, amino acids, peptides and glycopeptides, proteins, glycoproteins, etc. [17,18,19,20,21,22]. The effects of these active substances on human health have been proven, as they are included in the treatment of various diseases in humans [23]. For example, the regenerative effect of *Helix aspersa* Müller mucilage extract accelerates wound healing [24,25,26] on human dermal fibroblasts (MRC-5) [27] and on human keratinocytes [28]. Also, the reducing and stabilizing role of the mucus of the garden snail *H. aspersa* in AgNP synthesis has been established [29].

A number of studies in recent years have shown that copper oxide nanoparticles (CuONPs) can successfully find application as antioxidant, antibacterial, and antifungal agents. An analysis conducted of the literature data, presented by Gudkov et al., 2024, shows that copper peroxide nanoparticles have significant bacteriostatic potential against a wide range of bacterial strains and also contribute to the inhibition of the growth of some fungal strains. It was found that Gram-positive (Gram^+^) bacteria are more sensitive to the effect of CuONPs than Gram-negative (Gram^─^) bacterial strains. The mechanism of antibacterial activity of CuONPs is believed to involve adhesion to the microbial cell wall induced by electrostatic interactions. The dissociation of Cu^2+^ induces the generation of ROS. These ions also have the ability to enter cells, causing membrane and double-stranded DNA damage, which eventually causes cell death [30].

Pitt et al. reported the antibacterial activity of *C. aspersum* mucus, but mainly on *P. aeruginosa* [31] and on *S. aureus* [32]. However, the detectable antimicrobial properties of snail mucus could be due to the hindered diffusion of antimicrobial peptides when using the agar well method.

Recently, we published a paper on the green synthesis of copper oxide nanoparticles (CuONPs) formed from the active substances in the mucus of the garden snail *C. aspersum*, which exhibit antibacterial activity [3]. We presented two proteins from *C. aspersum* snail mucus with molecular weights (MWs) of 30.691 kDa and 26.549 kDa, which are reducing agents in the formation of CuONPs-Muc.

CuONPs are oligodynamic metals with the greatest application in medicine, diagnostics, antiaging therapies, etc. [33]. Therefore, the aim of this work was to develop a new method for the reduction of Cu^2+^ ions in an aqueous solution in the presence of two reducing agents: *C. aspersum* snail mucus and ascorbic acid in different concentrations. The advantage of the synergistic effect of the two reducing agents and the different concentrations of ascorbic acid on the production of CuONPs as well as their structure and antibacterial activity are also presented.

## 2. Results

### 2.1. Green Synthesis of CuONPs-Muc AsA

#### 2.1.1. Isolation and Characterization of a Mucus Extract from the Garden Snail *C. aspersum*

In this study, the extract was obtained via a patented technology [34] from the mucus of garden snails *C. aspersum*, grown in Bulgarian eco-farms. The assayed fraction was concentrated with a 20 kDa membrane to yield proteins with a molecular weight (MW) greater than 20 kDa [3]. The total protein concentration of the sample was determined by the Bradford, Lowry, and BCA methods, measuring the absorbance at 280 nm [35]. An isolated fraction from mucus extract with an MW > 20 kDa with a total protein concentration of 1.3 mg/mL was used in the green synthesis of CuONPs-MucAsA.

#### 2.1.2. Green Synthesis of CuONPs in Mucus Matrix with MW > 20 kDa and L-Ascorbic Acid

The green synthesis of CuONPs-Muc was carried out according to the method described by Dolashka et al., 2024 [3], after gradually adding 100 mL of 0.1 M CuSO_4_.5H_2_O to 100 mL of snail mucus fraction with an MW > 20 kDa under stirring at room temperature. CuONPs-MucAsA with two reducing agents—mucus extract with an MW >20 kDa and L-ascorbic acid—were synthesized using the same method.

The size and shape of CuONPs depend on various factors, such as the synthesis duration. Therefore, the effect of the added reducing component AsA on the reduction of Cu^2+^ ions and the mucus fraction followed after 1 h and 4 h.

After synthesizing CuONPs-Muc by the aqueous reduction of the mucus fraction at a pH of 7.0, a color change in the CuSO_4_.5H_2_O solution from dark blue to brown was observed at different concentrations of AsA (0.5 M and 1.0 M). The higher concentration of AsA aided in the faster formation of CuONPs after stirring the mixture for 4 h. This leads to a change in the color of the CuSO_4_.5H_2_O solution, which by the 4th hour becomes more intense due to the formation of CuONPs. After centrifugation at 4000 rpm and washing three times with water and acetone, the CuONPs were dried at 70 °C for 12 h. The re-dispersion procedure involves dissolving dried nanoparticles in d.H_2_O at a concentration of 0.020 mg/mL, followed by homogenization by vortexing for 5 min.

The three types of nanoparticles (CuONPs-Muc, CuONPs-Muc 0.5 M AsA, and CuONPs-Muc 1.0 M AsA) were obtained via different methods and techniques. Their physicochemical properties were investigated by observing their appearance and analytical methods, such as fluorescence, UV-Vis and infrared spectroscopy (FT-IR), scanning electron microscopy (SEM), thermogravimetric analyses (TG-DSC), the X-ray diffraction (XRD) technique, etc.

### 2.2. Characterization of the Obtained CuONPs-MucAsA

#### 2.2.1. UV–Vis and Fluorescence Analyses of CuONPs

**Figure 1:**
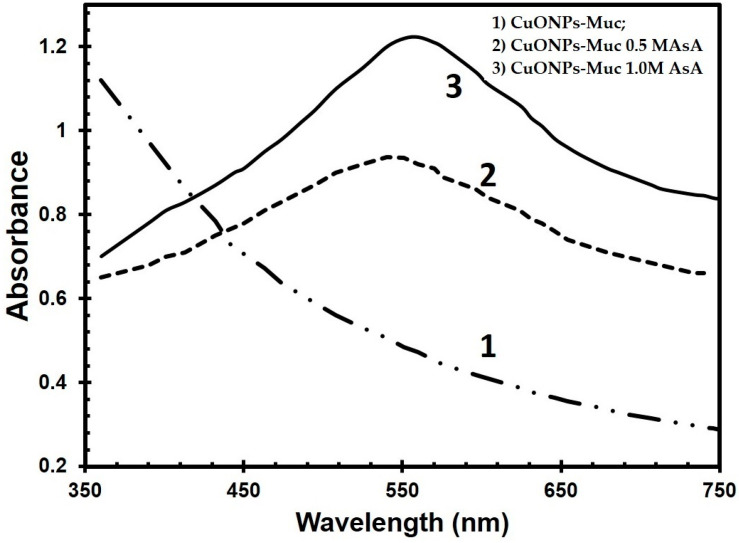
UV-Vis spectra of synthesized CuONPs after 4 h of incubation of the fraction with an MW > 20 kDa from snail *C. aspersum* and different concentrations of ascorbic acid, centrifuged at 4000 rpm: (1) CuONPs-Muc, (2) CuONPs-Muc 0.5 M AsA, and (3) CuONPs-Muc 1.0 M AsA.

The influence of different concentrations (0.0 M, 0.5 M, and 1.0 M) of AsA on the synthesis of CuONPs is expressed in a change in the UV-Vis spectra of the samples after 1 h and 4 h of incubation of the fraction with an MW > 20 kDa from the mucus, AsA, and CuSO_4_.5H_2_O. UV-Vis spectra confirmed the formation of CuONPs after 4 h of incubation of the mucus and 1.0 M AsA, represented by the more pronounced maximum at 560 nm in spectrum 3 (Figure 1) compared to the recorded spectrum 2 of the green synthesis in the presence of 0.5 M AsA and spectrum 1 of the green synthesis without AsA.

**Figure 2:**
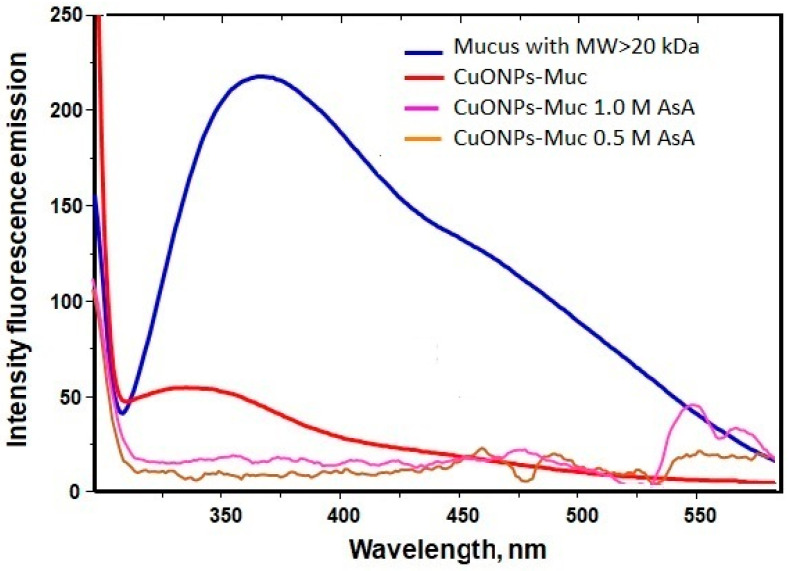
UV emission spectra of the mucus fraction with an MW > 20 kDa from snail *C. aspersum* (A280 = 0.1 in 0.1 M Tris buffer, pH 8) and synthesized CuONPs at different concentrations of ascorbic acid: the fraction with an MW > 20 kDa (blue line); CuONPs-Muc (red line); CuONPs-Muc 0.5 M AsA (purple line), and CuONPs-Muc 1.0 M AsA (brown line). Emission spectra were recorded with a 1 cm quartz cuvette after excitation at 295 nm.

The synthesis of CuONPs-Muc and CuONPs-MucAsA was also followed by fluorescence spectroscopy, a much more sensitive method. The obtained emission spectra in the range 305–650 nm after excitation at λ_ex_ 295 nm originated only from the emission of tryptophan (Trp) residues in the polypeptide chains of the mucus proteins. Analyses of the emission spectra of the four samples (Figure 2) show a change in both the position of the fluorescence emission maximum (λ_max_) (from 360 nm to 335 nm, respectively) and the emission intensity. The observed blue shift of the emission maximum from 360 nm, as well as the decrease in intensity, indicates that the formation of CuONPs-MucAsA is accompanied by a change in the conformation of the proteins in the corona of the biogenic nanoparticles. On the other hand, the presence of AsA, as an additional Cu2+-reducing agent is responsible also for encapsulation of the biogenic CuONPs-MucAsA.

#### 2.2.2. Characterization of the CuONPs-Muc AsA by Scanning Electron Microscopy Combined with Energy-Dispersive Spectroscopy (SEM/EDS)

Various SEM, EDX, and XRD investigations were carried out to evaluate the surface morphology and core mapping of the produced CuONPs. Based on the established morphology of CuONPs-Muc and the information from scanning electron microscopy with energy-dispersive spectroscopic analyses, the characteristics of the synthesized CuONPs were elucidated.

**Figure 3:**
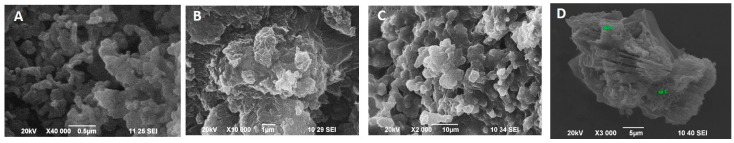
SEM images of CuONPs-Muc with reducing agent: (**A**) mucus fraction with MW > 20 kDa; (**B**) two reducing compounds—mucus fraction with MW > 20 kDa and 0.5 M AsA; (**C**) mucus fraction with MW > 20 kDa and 1.0 M AsA; and (**D**) 1.0 M AsA only.

The SEM/EDS images (JEOL JSM6390 and Oxford Instruments) shown in Figure 3 reflect the surface morphology of the CuONPs obtained at different concentrations of ascorbic acid. Only a few spherical particles synthesized in the presence of only the fraction with an MW > 20 kDa were observed (Figure 3A), while the best synthesis was achieved after mixing the two reducing agents: the mucus fraction with an MW > 20 kDa and 1.0 M AsA (Figure 3C). As shown in Figure 3D, the AsA solution alone did not induce the synthesis of CuONPs under identical green synthesis conditions, while the reduction of copper ions after the addition of 0.5 M AsA to the mucus fraction with an MW > 20 kDa was weaker (Figure 3B) compared to the synthesis of CuONPs-MucAsA in the presence of 1.0 M AsA (Figure 3C).

**Figure 4:**
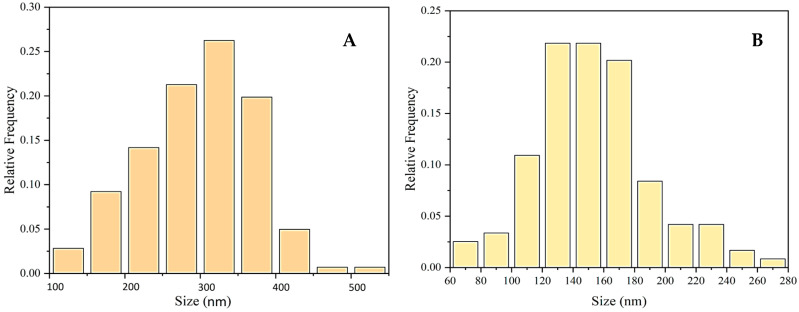
Histograms of particle size distribution of CuONPs-Muc obtained with protein mucus fraction with MW > 20 kDa as a reducing agent (**A**) and the same fraction in the presence of 1.0 M AsA (**B**).

The nanoparticles obtained using the mucus fraction as a reducing agent have a predominant size distribution between 250 and 400 nm (Figure 4A), while the particles formed from the same fraction in the presence of 1.0 M AsA have significantly smaller sizes ranging mainly from 130 to 170 nm, as shown in Figure 4B.

Energy-dispersive X-ray spectroscopy (EDS) is a non-destructive method that is commonly used in conjunction with scanning electron microscopy (SEM). EDS analysis enables the identification and quantification of the elemental composition of a sample at the macro-, micro-, or nanoscale.

**Figure 5:**
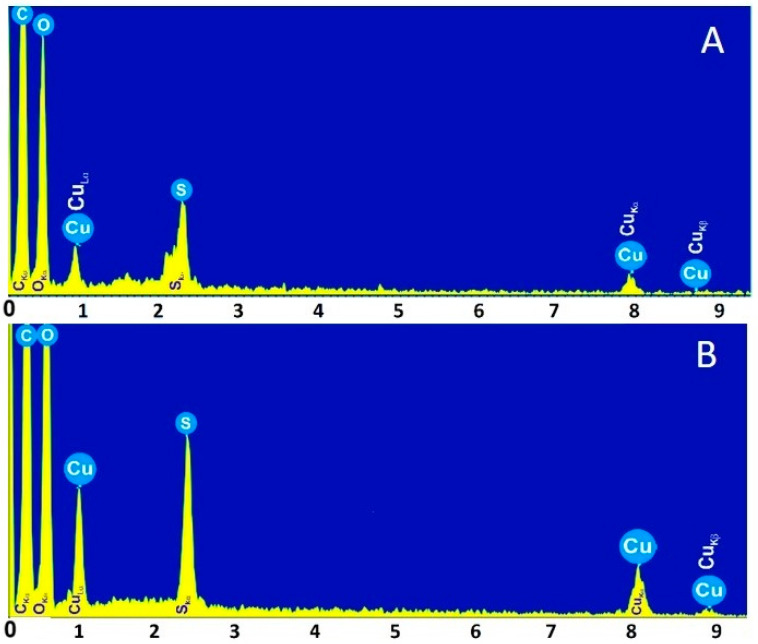
EDS analyses of CuONPs-Muc synthesized using two reducing agents of Cu^2+^ ions: (**A**) mucus fraction with MW > 20 kDa and 0.5 M AsA; (**B**) same protein fraction and 1.0 M AsA.

A comparative study of the EDS spectra of Cu/Cu_2_O nanoparticles shows the influence of AsA on CuONP synthesis. EDS analysis reported a higher amount of copper (Cu) obtained, which reflects the purity of the synthesized CuONPs-MucAsA in the presence of 1.0 M AsA (Figure 5).

Table 1 shows that the mucus fraction with an MW > 20 kDa is rich in various elements, such as C, O, S, Cu, Cl, Ca, Mg, Na, etc., with C and O being predominant. Green synthesis caused an increase in Cu concentration, with the highest value obtained in the presence of 1.0 M AsA, the increase being about 2-fold.

#### 2.2.3. Characterization of CuONPs-Muc AsA by X-Ray Diffraction (XRD) Technique

**Figure 6:**
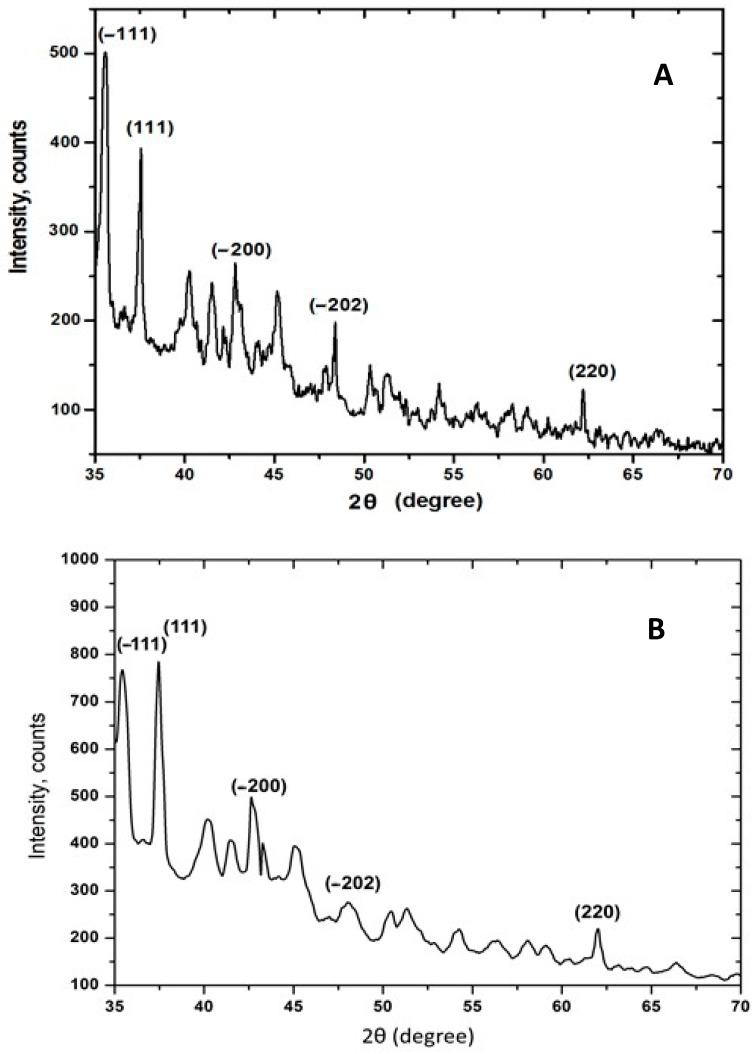
XRD analysis of CuONPs-MucAsA, obtained in the presence of (**A**) 0.5 M AsA and (**B**) 1.0 M AsA.

The X-ray diffraction results show that the nanoparticles formed by reducing Cu^2+^ ions from the mucus fraction with an MW >20 kDa in the presence of AsA are crystalline. The characteristic Bragg peaks, representative at 2θ = 35.6°, 2θ = 37.6°, and 2θ = 48.39°, indicate the crystallographic planes (−111), (111), and (−202) of CuO and confirm the formation of copper oxide in the nanostructured particles (Figure 6). Further evidence is provided by other diffraction peaks at 2θ = 42.82° and 2θ = 62.20°, characterizing the crystallographic planes (−200) and (220), corresponding to the cubic phase of Cu_2_O (ICDD: number 98-015-0886). On the other hand, the observed XRD noise is likely due to proteins and glycoproteins in the mucus matrix, which may be responsible for reducing copper ions. Thus, the presented XRD pattern clearly illustrates that the copper nanoparticles formed in the mucus matrix in the presence of AsA are crystalline.

Comparative analysis of the XRD diffractograms of the NPs obtained in the mucus fraction with an MW > 20 kDa with 0.5 M AsA (Figure 6A) and 1.0 M AsA (Figure 6B) shows the presence of peaks in both, indicating the formation of crystals. However, using 1.0 M AsA, the peaks are more pronounced, indicating the formation of more CuONP crystals. This proves that the synergistic effect of 1.0 M AsA and the mucus fraction with an MW > 20 kDa are the best reducers of copper ions.

#### 2.2.4. Characterization of CuONPs Using Fourier Transform Infrared Spectroscopy (FT-IR)

**Figure 7:**
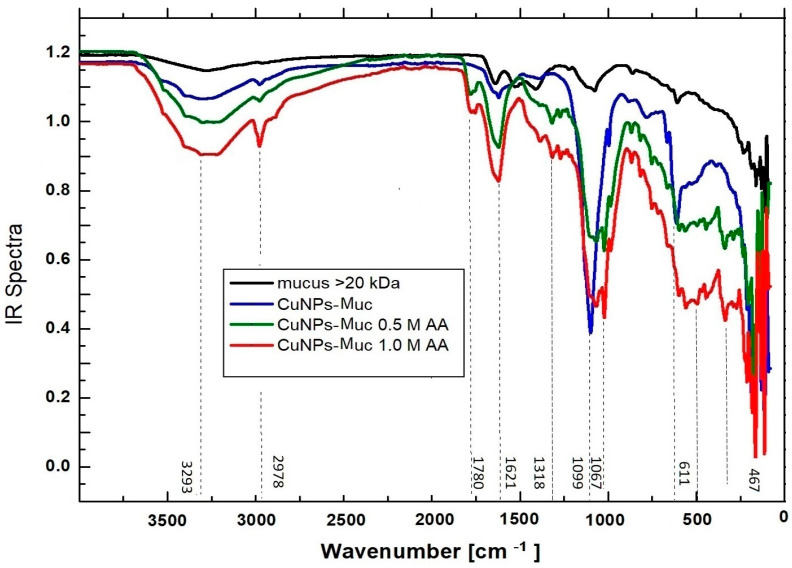
Infrared spectra of the mucus fraction with an MW > 20 kDa (black) and the formed CuONPs-Muc (blue line); CuONPs-Muc 0.5 M AsA (green); and CuONPs-Muc 1.0M AsA (red).

Characterization of the formed CuONPs-Muc without and in the presence of ascorbic acid was carried out by FT-IR. The recorded FT-IR spectra of the native mucus fraction with an MW > 20 kDa and CuSO_4_.5H_2_O that synthesized CuONPs-Muc and CuONPs-MucAsA in the presence of 0.5 M and 1.0 M AsA (Figure 7) showed that proteins in the mucus fraction are involved in the synthesis of CuONPs-Muc.

**Figure 8:**
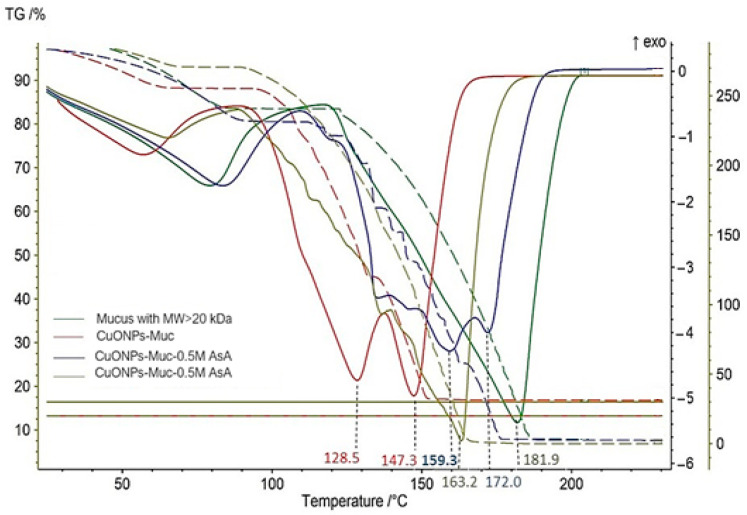
TG–DSC characteristics of pure mucus fraction with MW > 20 kDa (olive-green line); CuONPs-Muc (red line); CuONPs-Muc 0.5 M AsA (blue; line); and CuONPs-Muc 1.0 M AsA (green line). TG (dotted line) and DSC (solid line).

The comparison of the infrared spectra of the different samples presented in Figure 8 shows differences in the vibrational bands between 500 cm^−1^ and 4000 cm^−1^ and, correspondingly, the changes in the functional groups under different conditions.

Figure 8 presents the important absorption bands in the FT-IR spectrum of the investigated nanocomposite before and after the green synthesis of Cu/Cu_2_O NPs with the following reducing reagents: snail mucus with an MW >20 kDa and CuONPs-MucAsA with two concentrations of AsA at 467, 597, 611, 1022, 1067, 1099, 1318, 1621, 1780, 2978, and 3293 cm^−1^.

They are related to Cu–O, C–C, C–H, C=O, O–H, and Cu(II)–O [Table 2] [36,37,38,39] and correspond to the presented stretching vibration of CuO tabulated for other proteins reported by Barth et al., 2007 [40].

#### 2.2.5. Stability of CuONPs-Muc Analyzed by Thermogravimetric Analyses (TG/DSC-MS)

Information on the interactions between the protein (mucilage) and the copper particles, as well as the thermal stability of the composites, is provided by the thermogravimetric analyses with TG-DSC, where the TG curves reflect the changes in sample weight with increasing temperature and the DTG curves show the ongoing processes over time while heating the material.

The results of the TG-DSC analyses presented in Figure 8 report a large difference in the thermal degradation processes of the pure mucus fraction with an MW > 20 kDa and the formed CuONPs-MucAsA in the presence of 0.5 M and 1.0 M AsA.

Two clearly distinguished stages take place while heating in the range 25–300 °C, in an argon atmosphere, in the studied samples. The first stage ranges from 25 to 120 °C, with endothermic effects at around 60–80 °C and mass loss corresponding to the evaporation of physically adsorbed water. Important parameters were determined for the starting mucus fraction with an MW > 20 kDa (T_max, IDS_ = 73.3 °C, M_loss, IDS_ = 16.94%), for the synthesized CuONPs-Muc composites (T_max, IDS_ = 62.2 °C, M_loss, IDS_ = 23.47%), for CuONPs-Muc 0.5 M AsA (T_max, IDS_ = 63.2 °C, M_loss, IDS_ = 20.91%), and for CuONPs-Muc 1.0M AsA (T_max, IDS_ = 88.6 °C, M_loss, IDS_ = 16.37%) [Table 3].

In all four samples, the endothermic peak due to the release of water is followed by an exothermic peak that is much more pronounced in the native mucus and located at higher temperatures (T_peak_ = 135.8 °C) compared to CuONPs-Muc (T_peak_ = 91.5 °C), CuONPs-Mus 0.5 M AsA (90.7 °C), and CuONPs-Muc 1.0 M AsA (T_peak_ = 118.5 °C).

The second stage in the temperature ranges from 120 to 300 °C, characterized by significant mass loss in all samples (mucus fraction with MW > 20 kDa − M_loss1_ = 75.75%; CuONPs-Muc − M_loss1_ = 69.44%; CuONPs-Muc 0.5 M AsA − M_loss1_= 70.21%; CuONPs-Muc 1.0 M AsA − M_loss1_ = 72.84%), is due to the initial degradation of the protein molecules, and the complete destruction of the material occurs above 180 °C [Table 3].

The influence of the added 0.5 M and 1.0 M AsA on the intramolecular interactions in the nanoparticles was monitored. The observed differences in the compared values for the enthalpy (∆H) of CuONPs-Muc, CuONPs-Muc 0.5 M AsA, and CuONPs-Muc 1.0 M AsA are presented in Table 4.

### 2.3. Antibacterial Activity of CuONPs-MucAsA

**Figure 9:**
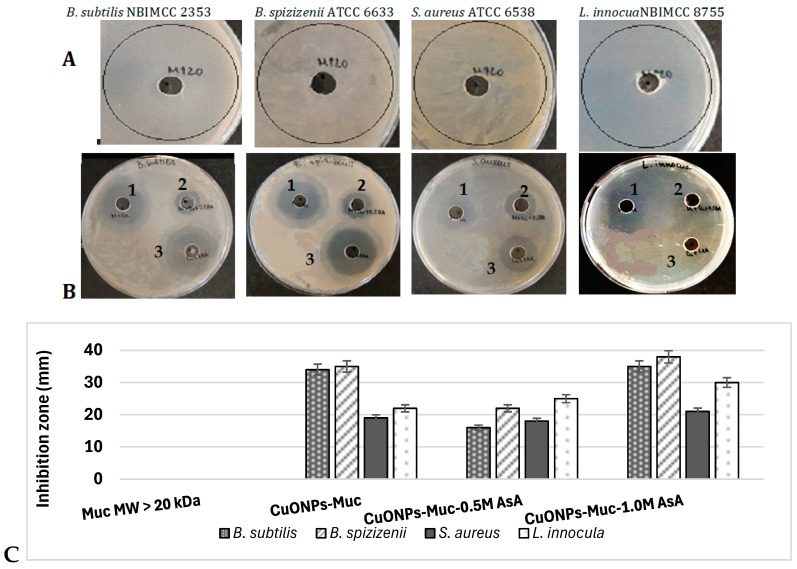
Antimicrobial effect against Gram^+^ bacterial strains of (**A**) mucus fraction from *C. aspersum* with MW > 20 kDa; (**B**) CuONPs synthesized with reducing agents (1) CuONPs-Muc, (2) CuONPs-Muc 0.5 M AsA, and (3) CuONPs-Muc 1.0 M AsA; (**C**) Comparative analysis between inhibitory zones of mucus fraction from *C. aspersum* with MW > 20 kDa and different synthesized CuONPs against Gram^+^ test bacterial strains.

**Figure 10:**
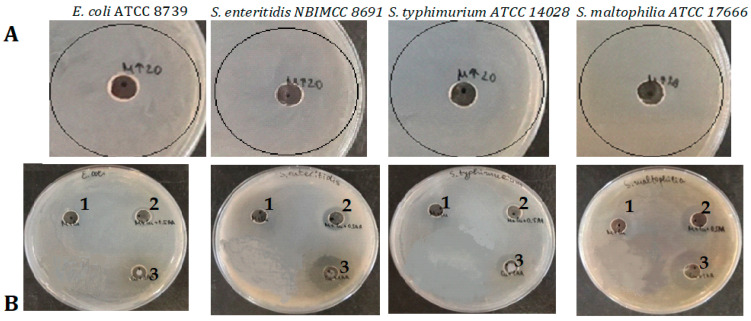

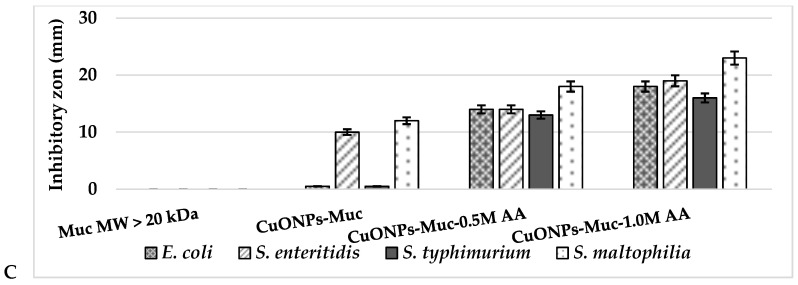
Antimicrobial effect against Gram^−^ bacterial strains of (**A**) mucus fraction from *C. aspersum* with MW > 20 KDa; (**B**) CuONPs synthesized with reducing agents (1) CuONPs-Muc, (2) CuONPs-Muc 0.5 M AsA, and (3) CuONPs-Muc 1.0 M AsA; (**C**) Comparative analysis between inhibitory zones of pure mucus of *C. aspersum* and different synthesized CuONPs against Gram^−^ test bacterial strains.

The antibacterial efficacy and spectrum of action of different variants of CuONPs was tested against four Gram-positive (*B. subtilis, B. spizizenii, S. aureus,* and *L. innocula*) and four Gram-negative (*E. coli*, *S. enteritidis*, *S. typhimurium,* and *S. maltophilia*) bacterial strains using the well diffusion method. The inhibitory effect of the synthesized CuONPs-MucAsA was compared with the mucus fraction from *C. aspersum* with an MW > 20 kDa and CuONPs-Muc. The obtained data revealed that the mucus fraction with an MW > 20 kDa had no detectable antibacterial effect on any of the eight bacterial strains used (Figure 9A and Figure 10A). However, when it was used as a reducing agent for the formation of CuONPs alone or in combination with ascorbic acid, significant antimicrobial properties emerged. Against Gram^+^ bacterial strains, the highest inhibitory effect was reported for CuONPs-Muc and CuONPs-Muc 1.0 M AsA, the zones of which reached 35–38 mm for *B. subtilis* and *B. spizizenii*.

A 50% lower growth inhibition was found in *S. aureus* and *L. innocula* for the same nanoparticles. The antibacterial activity of the nanoparticles synthesized by combined reduction with snail mucus and 0.5 M AsA was relatively weaker, with the inhibitory zone varying between 16 and 22 mm (Figure 9B,C).

Analyses of the antimicrobial activity of the tested CuONPs on the growth of Gram^−^ bacterial strains showed a different trend. In general, the effect of the synthesized nanoparticles was significantly weaker against Gram^−^ bacterial strains compared to the tested Gram^+^ bacterial cultures. Furthermore, the most effective in inhibiting the microbial growth of Gram^−^ strains was the CuONPs synthesized with the addition of the reducing agent AsA, with the zone of inhibition varying between 16 and 23 mm. At the same time, the obtained CuONPs-Muc from mucus alone had practically no effect against *E. coli* and *S. typhimurim* and was very weak against *S. enteritidis* and *S. maltophilia* (10–12 mm) (Figure 10B,C).

## 3. Discussion

In recent years, the biogenic synthesis of metal nanoparticles has been actively developing, but reports on the use of extracts of animal origin (such as body fluids) for the synthesis of nanoparticles compared to plant and microbial materials are very few [17]. Several studies have reported metal nanoparticles produced by green synthesis in a land snail mucus matrix [3,15,41,42,43].

The main objective of this study was focused on the use of two reducing agents—mucus of the garden snail *C. aspersum* with an MW > 20 kDa and L-ascorbic acid—to synthesize CuONPs-MucAsA to obtain better physicochemical characteristics, applying two main approaches.

The first approach reflects the synergistic effect of two reductants—the mucus of the garden snail with an MW > 20 kDa and L-ascorbic acid—on the formation of CuONPs-MucAsA, which is based on an ecological method published by us for the synthesis of biogenic CuONPs-Muc from the mucus of the garden snail *C. aspersum* with an MW > 20 kDa [3] and on literature data on the preparation of CuONPs with ascorbic acid [11]….

The second approach followed the influence of AsA concentration on CuONPs-MucAsA synthesis and included several experiments with a reducing agent—snail mucus with an MW > 20 kDa—and different concentrations of AsA (0.0 M, 0.5 M, and 1.0 M) to find the best biogenic pathway for producing copper nanoparticles.

Usually, some compounds such as polyethylene glycol and polyvinylpyrrolidone are used to prevent agglomeration in NP synthesis, but in the present study, this role is performed by AsA, which also serves as a capping agent [44,45]. The mechanism of synthesis of biogenic metal nanoparticles consists of three main phases: (1) the activation phase—the reduction of metal ions; (2) the growth phase—small nanoparticles spontaneously aggregating into larger particles, and (3) the completion phase of the process, determining the final shape of the nanoparticles [46,47]. We propose that the effect of AsA on green synthesis is related to the activation phase and termination phase of the process. Therefore, the second approach aimed to synthesize CuONPs with the *C. aspersum* protein fraction with an MW > 20 kDa, but in the presence of 0.5 M and 1.0 M AsA, which acts as a reducing and stabilizing agent. 

The synergistic effect of these two reductants on the formation of CuONPs-MucAsA was confirmed by a comparative analysis of the results obtained from the research conducted on NPs using different methods and techniques.

Four important advantages of the synthesized nanoparticles in the presence of two reducing agents were demonstrated compared to using snail mucus alone.

The first important advantage is a significant shortening of the synthesis time of CuONPs-MucAsA in the presence of two reducing agents from 3 days to 4 h. The first indication in the green synthesis of the nanoparticles is the color change in the reaction mixture due to the ongoing reduction processes [48]. The comparative analysis confirmed that adding AsA at different concentrations (0.5 M and 1.0 M, respectively) affected the formation rate of the biogenic nanoparticles compared to the reaction mixture without AsA.

The size and shape of CuONPs depend on several factors, such as the concentration of the starting sample and copper ions, the synthesis duration, the medium’s pH, the temperature, etc. Biogenic synthesis performed at room temperature showed the formation of CuONPs-Muc after 3 days of incubation of the garden snail mucus fraction mixture with an MW > 20 kDa with 0.1 M CuSO_4_.5H_2_O solution. In contrast to this method, the addition of AsA significantly reduced the synthesis time from 3 days to 4 h. Confirmation of the acceleration of the synthesis in the presence of AsA is given by the absorption spectra shown in Figure 1 by observing the characteristic peak for CuONPs-MucAsA at 530 nm, which is more pronounced for the reaction mixture containing 1.0 M AsA. Several studies have shown the dual role of ascorbic acid as a reducing and stabilizing agent in the synthesis of gold, silver, and copper NPs [14,45,49].

The formation of copper nanoparticles in the presence of AsA was also proven by the results obtained using fluorescence spectroscopy. Interactions of amino acids such as proline (Pro), tryptophan (Trp), or tyrosine (Tyr) with hydrophobic regions in the secondary structures of proteins are known to be involved in the processes of nanoparticle preparation [50,51]. The observed changes in the emission spectra after excitation at 295 nm of the starting sample—the fraction of mucus with an MW > 20 kDa and after the synthesis of CuONPs-Muc in the presence of AsA—confirm the formation of CuONPs-MucAsA. The emission spectrum of the mucus fraction with an MW > 20 kDa shows a distinct maximum at 360 nm related to the emission of Trps exposed on the surface of the proteins found in the mucus. Recently presented studies show that proteins and glycoproteins, peptides, and amino acids are among the main components of snail mucus that are responsible for the formation of gold and silver nanoparticles [3,41,42].

The observed change in the emission of Trp residues after they fall into the more hydrophobic environment “buried” in the formed aggregates is confirmation of NP formation. They reflect the reorganization of the secondary structure and spatial conformation (three-dimensional conformation) of proteins as reducing and stabilizing agents of the formed CuONPs-MucAsA.

The second important advantage is obtaining well-formed and stable CuONPs-MucAsA and determining their physicochemical properties. The data obtained from the applied SEM, EDS, and XRD analyses provide evidence for the formation of nanoparticles and information about their physicochemical properties.

The reaction process of CuONPs formation with the reducing agent AsA was investigated [18] by following the changes in X-ray diffraction (XRD) patterns at different times during the reaction. It was found that Cu(OH)_2_ is initially formed from Cu^2+^, which is reduced to Cu_2_O by AsA. Cu_2_O is an intermediate component that forms Cu particles at the end of the process. Confirmation of these studies is the obtained SEM images and XRD patterns of the synthesized CuONPs-Muc in the presence of ascorbic acid and at a reaction pH of 7, and well-formed NPs are presented in Figure 3 and Figure 6. The obtained X-ray diffraction results show that the nanoparticles formed by the reduction of Cu^2+^ ions from the mucus fraction in the presence of AsA are crystalline in nature. The characteristic Bragg peaks representative at 2θ = 35.6°, 2θ = 37.6°, and 2θ = 48.39° indicate the crystallographic planes (−111), (111), and (−202) of CuO and confirm the formation of copper oxide in the nanostructured particles (Figure 6). Additional evidence includes other diffraction peaks at 2θ = 42.82° and 2θ = 62.20° characterizing the crystallographic planes (−200) and (220) and corresponding to the cubic phase of Cu_2_O. On the other hand, the observed noise in XRD is likely due to proteins and glycoproteins present in the mucus matrix, which may be responsible for the reduction of copper ions. Thus, the presented XRD pattern clearly illustrates that the copper nanoparticles formed in the mucus matrix in the presence of AsA are crystalline in nature. In addition to the characterized Bragg peaks, additional yet undefined peaks are also observed.

The comparative analysis of the surface of CuONPs-MucAsA was performed using SEM/EDS. The SEM/EDS images showed only a few spherical particles synthesized by mucus with an MW > 20 kDa as a reducing agent (Figure 3A) and poor synthesis in the presence of 0.5 M AsA (Figure 3B), and the best synthesis was achieved with 1.0 M AsA (Figure 3C). Important information about the formation of nanoparticles is provided by FT-IR studies that identify the molecules involved in the synthesis of CuONPs. The aggregation of NPs is the result of two types of interactions: between Cu ions and side groups of proteins, as well as between proteins themselves (Van der Waals interactions), electrostatic, covalent, and hydrogen bonds, and π–π arrangements [52]. Snail mucus contains active compounds such as metal ions—copper (Cu), iron (Fe), and zinc (Zn); vitamins A, C and E; polyphenols; glycolic, hyaluronic, and lactic acid; and allantoin—as well as natural peptides, collagen, elastin, proteins, and enzymes (superoxide dismutase (SOD) and glutathione S-transferase (GST)) [53]. The importance and involvement of proteins and enzymes in the creation and stability of MNPs has been demonstrated with fungal biomass filtrate via FT-IR analyses [54]. Also, the inclusion of some proteins and enzymes from the mucus of the garden snail *C. aspersa* in the green synthesis of NPs was reflected by the changes in functional groups on the surface of CuONPs-MucAsA [3].

The positions and intensities of the observed bands in the spectra change based on the synthesis process and the content of the reducing components of the mucus and ascorbic acid. The comparative analysis of the recorded FT-IR spectra of the starting mucus and of the resulting copper system synthesized with a reducing reagent—the proteins in the mucus fraction and ascorbic acid (CuONPs-Muc, CuONPs-Muc 0.5 M AsA, and CuONPs-Muc 1.0 M AsA)—reflects changes in functional groups through different vibrational bands in the interval between 500 and 4000 cm^−1^ [43,44]. The broad band in the FT-IR spectra with a maximum at 3251 cm^−1^, associated with the stretching vibrations of oxygen and hydrogen (in -OH), confirms the presence of an organic matrix around the copper core. The other band at 2924 cm^−1^ reflects carbon traces resulting from C-H stretching vibrations [55].

Important information is provided by the two peaks at 1780 and 1621 cm^−1^ reflecting the stretching vibrations of the C=O bond in the amides (amide I and amide II). The absorption at 1780 cm^−1^ accounts for the stretching vibrations of the C=O bond of amide I, while the absorption at 1621 cm^−1^ is mainly due to the bending vibrations of the NH bond of amide II. The observed change in intensity of both bands reflects an increase in protein content, which is most pronounced for CuONPs-Muc 1.0 M AsA.

Another strong band in the spectra of the samples is observed at 1099 cm^−1^, which represents the C-N stretching vibrations of the amines and carboxylate moieties associated with the acid derivatives of the sugars and amino acid chains [56]. They are a confirmation of the involvement of existing polysaccharides in the mucus in the formation of CuONPs-MucAsA. The C-C stretching and O-H bending vibrations are reflected by the band at 1022 cm^−1^. The absorption bands observed in the FT-IR spectra with a maximum at 467 cm^−1^, 597 cm^−1^, and 611 cm^−1^ (Table 2, Figure 7) confirm the green synthesis of CuO nanoparticles in a matrix of the protein fraction from the *C. apersum* mucus without and in the presence of AsA [40,41]. Comparative analysis of changes in the intensity and positions of the observed bands in the FT-IR spectra reported the most pronounced changes in the formed CuONPs-Muc 1.0 M AsA, which reflect the contribution of AsA to the formation and stabilization of well-formed CuONPs [56].

The third important advantage of CuONPs-MucAsA synthesized in the presence of two reducing agents is the higher conformational and thermal stability. Important information is also provided by the conformational and thermal stability of the obtained NPs, which is related to the proteins forming the agglomerates. Based on the published data, it has been suggested that the lower conformational stability of NPs in biological solutions and their greater thermal stability are related to the participation of mainly low-molecular-weight compounds in the formation of nanoparticles [3,57,58,59].

The comparative analysis of the values obtained from the TG and DSC analyses for the fraction with an MW > 20 kDa and the formed NPs (CuONPs-Muc, CuONPs-Muc 0.5 M AsA, and CuONPs-Muc 1.0 M AsA) clearly outline two stages in the process of heating in an argon atmosphere in the temperature range 25–300 °C. They are represented by the TG curves, which reflect changes in the weight of the sample, and the DTG curves related to the ongoing processes over time as a result of heating the material. The data for the formed CuONPs under different conditions show that the endo- and exo-processes take place at lower temperatures compared to the changes in the starting mucus fraction with an MW > 20 kDa. This is mainly due to the adsorption of low-molecular-weight compounds from the mucus in the CuONPs formed. This leads to their faster degradation.

Low-molecular-weight proteins are known to undergo weaker conformational changes than higher-molecular-weight proteins upon contact with the substrate surface [60]. Probably, the lower thermodynamic affinity and weaker conformational changes in the structure of low-molecular-weight proteins are the result of a smaller number of binding points on the protein molecule [52].

The importance of AsA in stabilizing the synthesized CuONPs-Muc is confirmed by the presented TG/DSC-MS analyses in Figure 9 and Table 3 and Table 4, which reflect a change in the thermal stability of CuONPs-Muc as well as the interactions between the protein and CuONPs-Muc. The higher conformational stability and stronger intramolecular interactions in high-molecular-weight proteins compared to low-molecular-weight proteins are reflected by the higher values of enthalpy (∆H), which are determined from the peak area of the DCS curves for the starting mucus fraction with an MW > 20 kDa (∆H = −1216 J/g). The addition of AsA leads to the strengthening of intramolecular interactions and therefore to the higher enthalpy values for CuONPs-Muc 0.5 M AsA and CuONPs-Muc 1.0 M AsA (ΔH = −1076 J/g and −1119 J/g, respectively) compared to CuONPs-Muc (ΔH = −1058 J/g) and to an increase in their thermal stability (T_peak_ = 159.3 °C and 163.2 °C, respectively). An increase in the thermal stability of the composites with 0.5 M and 1.0 M AsA is probably also because ascorbic acid coats the surface of CuONPs and accordingly increases their thermal stability.

The fourth important advantage of synthesized CuONPs-Muc in the presence of two reducing agents is more pronounced antibacterial activity against some Gram^+^ and Gram^−^ bacterial strains. CuONPs are known to be potent antimicrobial agents because microorganisms survive only a few minutes on their surface [61]. Recently, we showed promising antibacterial properties of CuONPs-Muc against several Gram+ and Gram^−^ bacteria [3]. Although pure mucus did not show antimicrobial properties against the tested strains, this does not exclude the possibility of the presence of antimicrobial proteins in its composition. Combinations of antimicrobial agents are known to have synergistic antimicrobial activity. Even an antimicrobial agent not active against a particular bacterial strain can enhance the activity of the second agent when combined [62].

Despite the established antibacterial effect for the four CuONPs-Muc variants, they exhibited more pronounced antibacterial activity against Gram^+^ strains compared to Gram^−^ strains. For example, in the case of Gram^+^ strains, the average values of the inhibitory zone reached 24.7 mm, and for Gram^−^ strains, they reached −13.7 mm. The most probable reason for this is the difference in the arrangement of the cell walls of Gram^+^ and Gram^−^ bacterial cells. Cell wall structure plays a key role in bacterial sensitivity or tolerance to metal NPs [63]. Gram^+^ bacteria have a thick layer of peptidoglycan in their wall, while Gram^−^ bacteria have a thinner peptidoglycan as well as an outer membrane rich in lipopolysaccharide (LPS). This outer membrane represents an additional protective barrier for Gram^−^ bacteria against the entry of nanoparticles. LPS can prevent the adhesion of NPs to the cell surface and regulate the entry of metal ions [64,65]. On the other hand, Gram^+^ bacteria have in their cell walls teichoic acids with multiple phosphate groups, which carry a negative charge; therefore, they can strongly bind positively charged metal nanoparticles [66,67]. Thus, teichoic acids in the wall of Gram^+^ bacteria provide a higher negative charge compared to Gram^−^ bacteria, resulting in a stronger interaction with positively charged NPs [64,67]. In addition, Gram^−^ bacteria can precipitate toxic metal ions in their periplasmic space in the form of insoluble or slightly soluble oxides, sulfides, etc. [68]. Another characteristic of the walls of Gram^−^ bacteria is the presence of porins and transporters in the outer membrane, through which the cell can release toxic metal ions, which increases the resistance of these microorganisms to metals and metal nanoparticles [68].

Many other studies have also reported stronger antibacterial activity by metal NPs against Gram^+^ bacteria [64,69]; Azam et al. reported that ZnONPs and CuONPs showed 16–24% and 28–33% stronger antibacterial activity against Gram^+^ strains (*B. subtilis and S. aureus*) than to Gram^−^ strains (*E. coli* and *P. aeruginosa*) using the agar well method [70]. Similarly, our investigation revealed that CuONPs synthesized with pure snail mucus was characterized with 80% higher effectiveness compared to Gram+ bacterial strains than to Gram^−^ strains. In turn, CuONPs obtained via the reduction of snail mucus and ascorbic acid again had a 30–45% stronger inhibitory effect against Gram+ bacteria.

It is important to note that the CuONPs synthesized with added AsA and mucus extract possess higher antibacterial activity. This is probably due to the reducing properties of both reagents leading to the formation of nanostructures with better characteristics. This was also demonstrated by Mane et al., and the AgNPs synthesized with snail mucus and ascorbic acid showed increased inhibition of microbial growth [16]. Also, our research reveals that the CuONPs synthesized with pure snail mucus are characterized by 80% higher efficiency against Gram^+^ bacterial strains than against Gram^−^ strains, against which they show very weak activity. On the other hand, the CuONPs obtained using two reducing reagents—snail mucus and ascorbic acid—showed 30–45% stronger inhibitory effect against Gram^+^ bacteria compared to Gram^−^ bacteria, towards which they also showed high activity.

The addition of AsA and mucus extract during the synthesis of CuONPs increases their antibacterial activity against Gram^−^ bacteria. This is probably due to the reducing properties of both reagents leading to the formation of nanostructures with better characteristics. This was also shown by Mane et al., with AgNPs synthesized from snail mucus and ascorbic acid, which possessed an increased inhibitory effect against microbial growth [20]. Furthermore, the synthesized CuONP-Muc 0.5 M AsA and CuONP-Muc 1.0 M showed better antibacterial effect against Gram^−^ bacterial strains compared to CuONP-Muc, which may be due to their specific structural features. The larger size of NPs with AsA may allow for easier penetration through the less compact envelope of Gram^−^ cells.

## 4. Materials and Methods

### 4.1. Sample Preparation

The crude mucus was extracted from garden snails (*C. aspersum*) grown in Bulgarian eco-farms using patented technology described in [17,34]. After purification, including a series of operations as described in Dolashki et al., 2020, and Velkova et al., 2024, the native extract was concentrated by ultrafiltration through membrane filters with a pore size of 20 kDa [17,19]. The obtained protein fraction was used in the green synthesis of CuONPs in the presence of L-ascorbic acid (AsA) as described in the following subsection. The protein concentration was merged according to the Bradford method [35].

### 4.2. Green Synthesis of CuONPs from Snail Mucus

Three different biogenic snail mucus matrix CuONPs (MW > 20 kDa fraction) were prepared by green synthesis under different conditions. CuONPs-Muc were prepared according to a recently described method by [3], after gradually adding 100 mL of 0.1 M CuSO_4_.5H_2_O (Sigma-Aldrich, Saint Louis, MO, USA) to 100 mL of the snail mucus fraction with an MW > 20 kDa (0.2 mg/mL, pH~7) with continuous stirring at room temperature for 3 days.

In order to optimize the method and obtain nanoparticles with better physicochemical characteristics and antimicrobial properties, CuONPs-Muc were prepared in the presence of 0.5 M and 1.0 M L-ascorbic acid (Sigma Aldrich), respectively. After 1 h, 100 mL of 0.5 M and 1.0 M L-ascorbic acid, respectively, were added to the reaction mixture containing the mucus fraction with an MW > 20 kDa (0.2 mg/mL) and CuSO_4_.5H_2_O, resulting in the pH of the mixtures changing to 3. Both reaction mixtures were incubated at room temperature (about 24 °C) with continuous stirring at 250 rpm for 4 h. The color change in the two reaction mixtures from sea blue to dark brown was monitored. This color change in the colloid is an indication of the reduction of Cu^2+^ and the formation of CuNPs-Muc. The formed precipitate was centrifuged at 4000 rpm for 10 min and washed with acetone and three times with water, until a neutral pH of the solution was achieved; after that, it was dried at 70 °C for 12 h. The re-dispersion of dried nanoparticles was conducted in d.H_2_O at a concentration of 0.020 mg/mL, followed by vortexing for 5 min.

### 4.3. Characterization of Copper Nanoparticles

Physicochemical properties of the synthesized CuONPs were studied through analytical methods and appearance observations including UV-Vis (Shimadzu™ UVmini-1240 Shimadzu Corporation, Kyoto, Japan), fluorescence spectroscopy (Shimadzu RF-6000 spectrofluorometer, Shimadzu Corporation, Kyoto, Japan), FT-IR (infrared (FT-IR) spectrometer INVENIO-R, Bruker, Karlsruhe, Germany), scanning electron microscopy combined with energy-dispersive spectroscopy (SEM/EDS) (JEOL JSM6390, Tokyo, Japan and Oxford Instruments, UK), thermal analysis (TG) and differential scanning calorimetry (DSC) (STA 449 Jupiter F3 Netzsch apparatus, Selb, Germany), and X-ray diffraction analysis (XRD) (PW1050 goniometer, Philips, Almelo, The Netherlands).

#### 4.3.1. Characterization of CuONPs by UV-Vis Spectroscopy

The absorption spectra of the mucus fraction with an MW > 20 kDa and obtained from its CuONPs without and in the presence of 0.5 M and 1.0 M AsA were measured in the range of 350–750 nm using a UV-Vis spectrometer (Shimadzu™ UVmini-1240) via symmetric quartz cuvettes (Quartz Glass High Performance 200 nm–2500 nm, Hellma^®^ absorption cuvettes) with a 10 mm optical length at room temperature (25 °C). Background correction for all spectra was made by subtracting the water absorption spectrum in the same conditions.

#### 4.3.2. Fluorescence Spectroscopy Analyses of CuONPs

The comparative analysis of fluorescence emission spectra of samples containing the mucus fraction with an MW > 20 kDa used for obtaining CuONPs-Muc at different conditions by green synthesis (CuONPs-Muc, CuONPs-Muc with 0.5 M AsA, and CuONPs-Muc with 1.0 M AsA) were conducted on a Shimadzu RF-6000 spectrofluorometer (Shimadzu Corporation, Kyoto, Japan) equipped with a xenon lamp as the excitation source, using a fluorescent cuvette with a path length of 10 × 10 mm (Hellma 101-QS, Floressan Quartz Küvet, Hellma USA, Inc., Clementon, NJ,USA), at band λ_ex_ = 3 nm and band λ_em_ = 20 nm. Emission spectra were recorded between 305 nm and 650 nm, after excitation at λ_ex_ 295 nm, with a step of 0.5 nm. The emission spectra of the initial protein mucus fraction with an MW > 20 kDa and samples containing the obtained biogenic CuONPs were compared.

#### 4.3.3. Characterization of CuONPs by Means of SEM

In order to reveal the individual morphology of the CuONPs, we used a scanning electron microscope to obtain images of both secondary and back-scattered electrons. Since the samples are not very conductive, they were gently covered with thin gold film in order to avoid sample charging.

The surface morphology of the CuONPs obtained at different conditions and their chemical composition were characterized in numerous different regions by means of scanning electron microscopy (JEOL JSM6390, Japan, and Oxford Instruments, AZtec software v5.1, Oxford, UK). Several tenths of the individual SEM images of every sample were used in order to construct the histograms of the size distributions of the CuONPs.

#### 4.3.4. X-Ray Diffraction Analysis (XRD)

X-ray diffraction is a non-destructive technique that provides detailed information about the crystallographic structure, chemical composition, and physical properties of a material. It is based on the constructive interference of monochromatic X-rays and a crystalline sample. X-ray diffraction analyses were performed by a Panalytical Empyrean diffractometer using Highscore Plus software v5.3.

#### 4.3.5. Characterization of CuONPs-Muc by FT-IR

Information about the functional groups’ bonding between CuONPs obtained at different conditions in the snail mucus matrix (fraction with an MW > 20 kDa) were obtained by means of the Fourier transform infrared (FT-IR) spectrometer INVENIO-R (Bruker) with a resolution of 2 cm^−1^. All FT-IR spectra were recorded and accumulated via 120 scans using an IRIS single-reflection ATR diamond crystal plate (PIKE Technologies, Fitchburg, WI, USA).

#### 4.3.6. Characterization of CuONPs by DTG

Thermal analysis (TG) and differential scanning calorimetry (DSC) were performed with an STA 449 Jupiter F3 Netzsch apparatus (Germany). The device has three holders for different measurements. It is equipped with a built-in scale with very high accuracy. It works with very small amounts: 10–25 mg. Its temperature range is from 25 to 1400 °C, and its heating rate is 0.001–50 K/min. In addition to the apparatus, an attachment (press) was purchased for working with liquid samples. It works with corundum crucibles (up to 1400 °C), Al crucibles (up to 600 °C), and platinum crucibles.

Thermal analyses of the materials CuONPs-Muc, CuONPs-Muc with 0.5 M AsA, and 1.0 M AsA and the mucus fraction with an MW > 20 kDa used to prepare the biogenic nanoparticles were conducted at a heating rate of 10 °K/min in an Ar atmosphere, with a flow rate of 30mL/min, at a temperature of 25 °C–300 °C, and a sample mass of 30–50 mg. Al cuvettes were used. The thermal behavior of a pure mucus sample was compared with the thermal behavior of the composites (CuONPs). Running phase transitions in these samples during heating to 300 °C were also studied.

### 4.4. Antimicrobial Activity of CuONPs-Muc

Eight bacterial strains (four Gram-positive and four Gram-negative) obtained from the American Type Culture Collection (ATSC, Manassas, VA, USA) and the National Bank for Industrial Microorganisms and Cell Cultures (NBIMCC, Sofia, Bulgaria) were used in the current investigation (Table 5). The growth of the tested microbial strains was performed at 37 °C for 24 h in standard mesopeptone agar (MPA) with the following composition (g/L): peptone—5, HM peptone B (beef extract)—1, yeast extract—2, sodium chloride—5, and agar—22. Final pH = 7.4 ± 0.2.

The antibacterial effect of the following samples was tested by the agar well diffusion method: mucus fraction from *C. aspersum* with an MW > 20 kDa (pure); CuONPs + mucus (CuONPs-Muc); CuONPs + mucus + 0.5 M ascorbic acid (CuONPs-Muc 0.5MAsA); CuONPs + mucus + 1.0 M ascorbic acid (CuONPs-Muc 1.0MAsA) in a final concentration of 0.02 mg/mL. Bacterial suspensions from cultures grown over 24 h were prepared with optical density OD _600_ = 0.1 (0.5 McFarland). Nutrient agar plates were inoculated with 100 μL of the bacterial suspensions by dense surface inoculation. Wells were cut in the agar, and 100 μL of pure mucus and each NP’s sample were dropped into the wells, and the Petri dishes were placed in a refrigerator (temperature 4 °C) for four hours. Then, the plates were incubated at 37 °C for 24 h. The antibacterial effect of the NPs was evaluated with respect to the measured zones of cell growth inhibition (d, mm). Three experiments were performed in duplicate, and the values were averaged.

## 5. Conclusions

In conclusion, this paper presents a new green synthesis method of forming CuONPs-MucAsA from *C. aspersum* garden snail mucus extract with an MW > 20 kDa and L-ascorbic acid acting as a reducing and stabilizing agent. Two main approaches were considered in this study: the synergy between reducing agents and the influence of different concentrations of L-ascorbic acid (0.0 M, 0.5 M, and 1.0 M) on the synthesis of CuONPs-MucAsA. Together, these approaches illustrate a highly efficient biogenic pathway for the formation of copper nanoparticles, paving the way for future advances in environmentally friendly nanoparticle production.

The characteristics of the synthesized CuONPs-MucAsA were analyzed using various techniques including UV-Vis and fluorescence spectroscopy, XRD, SEM/EDS, FT-IR, and TG-DSC. A number of main advantages of these methods for the synthesis of NPs have been demonstrated. The use of two reducing agents significantly reduced the synthesis time from three days to four hours, and well-shaped and stable nanoparticles were also formed.

Despite the established antibacterial effect for the four CuONPs-Muc variants, they showed more pronounced antibacterial activity against Gram^+^ strains compared to Gram^−^ strains. Furthermore, CuONPs-MucAsA showed greater antibacterial activity against Gram^−^ bacteria compared to the mucus fraction and CuONPs-Muc. It is assumed that this antibacterial effect against Gram^−^ strains, such as *E. coli* ATCC8739, *S. enteritidis* NBIMCC8691, *S. typhimurium* ATCC 14028, and *S. maltophilia* ATCC 17666, is due to the more compact envelope of Gram^−^ cells.

Continued research in this area will elucidate the mechanisms of antimicrobial action of the new biogenic nanoparticles by further investigating the cellular redox status, transcription levels, and apoptotic cell markers in the tested pathogens.

## Figures and Tables

**Table 1:** EDS analysis of the elemental composition of the starting mucus fraction and CuONPs-Muc obtained at different conditions by green synthesis.

Mucus Fraction with MW > 20 kDa									
Element	C	O	S	Cu	Cl	Ca	Mg	Na	Total
wt%	46.08	43.24	2.33	2	0.71	1.06	1.44	3.15	100
at %	56.55	38.5	1.03	0.47	0.28	0.38	0.84	1.95	100
CuONPs-Muc									
wt %	49.92	43.29	2.51	4.27	-	-	-	-	100
at %	59.31	38.61	1.12	0.96	-	-	-	-	100
CuONPs-Muc 0.5 M AsA									
wt %	42.47	50.32	2.67	4.53	-	-	-	-	100
at %	51.73	46.01	1.22	1.04	-	-	-	-	100
CuONPs-Muc 1.0 M AsA									
wt %	42.81	46.32	3.55	7.32	-	-	-	-	100
at %	53.31	43.31	1.66	1.72	-	-	-	-	100

**Table 2:** Characteristic peaks in FT-IR spectra of *C. aspersum* fraction and CuONPs-MucAsA.

Wavenumber [cm^−1^]	Interaction
467	with Cu–O stretching vibration
597	the Cu(II)–O bond in CuO [36]
611	stretching of Cu(I)–O in Cu_2_O particles [37]
1022	C–C stretching vibration and O–H bending vibration
1067	Cu(II)–O bond in CuO [36]
1099	C–N and C–H stretching
1318	C–H stretching
1621	O–H bending and NH bond
1780	C=O bond in CHO group amide I and amide II
2978	C–H stretching [38]
3293	O–H stretching [38]

**Table 3:** TG–DTG data for the pure mucus fraction with an MW >20 kDa and CuONPs: mucus with an MW >20 kDa, CuONPs-Muc, CuONPs-Muc 0.5 M AsA, and CuONPs-Muc 1.0 M AsA.

Sample	TG				DTG		
	M_loss IDS_ [%] 30–120 [°C]	M_loss1_ [%] 120–200 [°C]	M_loss2_ [%] 200–300 [°C]	M_lossTOTAL_ [%]	T_max,IDS_ [°C]	T_max1_ [°C]	T_max2_ [°C]
Mucus with MW > 20 kDa	16.94	75.75	0.03	92.72	73.3	123.0	183.0
CuONPs-Mucus	23.47	69.44	0.04	92.95	62.2	134.9	160.9
CuONPs-Muc 0.5 M AsA	20.91	70.21	0.20	91.31	63.2	151.5	170.4
CuONPs-Muc 1.0 M AsA	16.37	72.84	1.27	90.48	88.6	120.3	177.2

*M_loss IDS_*, mass loss in initial decomposition step; *M_loss_*, mass loss in main decomposition step, *M_loss, TOTAL_*, total mass loss; *T_max_,_IDS_*, maximum temperature in initial decomposition step; *T_max 1,2_*, temperature in maximum degradation step.

**Table 4:** DSC data for mucus fraction with MW >20 kDa and CuONP composites.

Sample	DSC							
	T_onset_ [°C]	T_peak_ [°C]	T_end _ [°C]	∆H J/g	T_onset_ [°C]	T_peak_ [°C]	T_end _ [°C]	∆H J/g
Mucus with MW > 20 kDa	58.2	80.8	91.1	−289.2	127.9	181.9	189.0	−1216
CuONPs-Mucus	41.1	65.2	85.4	−183.8	125.3	147.3	170.7	−1058
CuONPs-Muc 0.5 M AsA	42.3	57.2	74.7	−139.3	130.0	159.3	185.6	−1076
CuONPs-Muc 1.0 M AsA	58.7	83.6	99.6	−119.6	106.4	163.2	175.4	−1119

*T_onset_*, temperature of decomposition initiation; *T_peak_*, peak maximum decomposition temperature; *T_end_*, final decomposition temperature; ∆*H*, heat generated during the decomposition reaction obtained by the integration of the thermal peaks (area).

**Table 5:** Test bacterial strains used.

Taxonomic Affiliation	Culture Collection	Number
**Gram-positive**		
*Bacillus subtilis*	NBIMCC	2353
*Bacillus spizizenii*	ATCC	6633
*Staphylococcus aureus*	ATCC	6538
*Listeria innocua*	NBIMCC	8755
**Gram-negative**		
*Escherichia coli*	ATCC	8739
*Salmonella enteritidis*	NBIMCC	8691
*Salmonella typhimurium*	ATCC	14,028
*Stenotrophomonas maltophilia*	ATCC	17,666

## Data Availability

Data are contained within the article.
